# Human Leukocyte Antigen-A, B, C, DRB1, and DQB1 Allele and Haplotype Frequencies in a Subset of 237 Donors in the South African Bone Marrow Registry

**DOI:** 10.1155/2018/2031571

**Published:** 2018-04-23

**Authors:** Mqondisi Tshabalala, Charlotte Ingram, Terry Schlaphoff, Veronica Borrill, Alan Christoffels, Michael S. Pepper

**Affiliations:** ^1^Department of Immunology, SAMRC Extramural Unit for Stem Cell Research and Therapy, Institute for Cellular and Molecular Medicine, Faculty of Health Sciences, University of Pretoria, Pretoria, South Africa; ^2^South African Bone Marrow Registry (SABMR), P.O. Box 13353 Mowbray, Cape Town 7705, South Africa; ^3^Laboratory for Tissue Immunology, National Health Laboratory Service, Groote Schuur Hospital, C21 Anzio Street, Observatory, Cape Town 7925, South Africa; ^4^SA MRC Bioinformatics Unit, South African National Bioinformatics Institute, University of Western Cape, Robert Sobukwe Road, Bellville, Cape Town 7535, South Africa

## Abstract

Human leukocyte antigen- (HLA-) A, HLA-B, HLA-C, HLA-DRB1, and HLA-DQB1 allele and haplotype frequencies were studied in a subset of 237 volunteer bone marrow donors registered at the South African Bone Marrow Registry (SABMR). Hapl-o-Mat software was used to compute allele and haplotype frequencies from individuals typed at various resolutions, with some alleles in multiple allele code (MAC) format. Four hundred and thirty-eight HLA-A, 235 HLA-B, 234 HLA-DRB1, 41 HLA-DQB1, and 29 HLA-C alleles are reported. The most frequent alleles were A∗02:02g (0.096), B∗07:02g (0.082), C∗07:02g (0.180), DQB1∗06:02 (0.157), and DRB1∗15:01 (0.072). The most common haplotype was A∗03:01g~B∗07:02g~C∗07:02g~DQB1∗06:02~DRB1∗15:01 (0.067), which has also been reported in other populations. Deviations from Hardy-Weinberg equilibrium were observed in A, B, and DRB1 loci, with C~DQB1 being the only locus pair in linkage disequilibrium. This study describes allele and haplotype frequencies from a subset of donors registered at SABMR, the only active bone marrow donor registry in Africa. Although the sample size was small, our results form a key resource for future population studies, disease association studies, and donor recruitment strategies.

## 1. Introduction

The ~4 Mb human leukocyte antigen (HLA) complex on chromosome 6 in humans is amongst the most polymorphic gene regions in the genome [1]. Seventeen thousand eight hundred and seventy-four (17874) HLA alleles have been described in the IMTG/HLA database to date [2]. HLA gene products are key for antigen presentation to T cells and form the basis of host defense mechanisms against pathogens [3]. HLA also plays a role in vaccine development and has a determining role in transplantation outcome [4–11]. In hematopoietic stem cell transplantation (HSCT), good clinical outcomes are associated with high-resolution HLA matching [12, 13], with the number of mismatches correlating with the risk of rejection and/or graft versus host disease (GVHD) [14–16].

Bone Marrow Donors Worldwide (BMDW) is a centralized databank of HLA phenotypes and other relevant data of unrelated stem cell donors which aims to support HSCT programmes [17]. The South African Bone Marrow Registry (SABMR), a nonprofit initiative based in Cape Town, was started in 1991 with the objective of providing HLA-matched unrelated donors for South African patients and the world at large. The registry, listed in the BMDW, has more than 73,000 HLA-typed volunteer donors from South Africa [18]. Unrelated donor registries globally, including the SABMR, increase chances of HLA matches for many patients in need of transplantation. Despite the high-donor numbers globally, it is still difficult to find HLA matches for patients of Black African origin, partly because of (a) the great genetic diversity in these populations [19] and (b) limited information on HLA diversity [9]. Most transplants facilitated by the SABMR are from foreign donors, mainly due to the limited number of donors in the registry, particularly those of Black African and Asiatic/Indian origin [20]. There is thus a need to improve recruitment from these underrepresented populations into the SABMR, which, since 1997, has been the only registry on the African continent supporting an HLA-matched unrelated donor stem cell transplantation programme [20, 21].

Donor registries continuously try to improve their recruitment strategies through increasing donor numbers [22], recruiting young males [23], minority recruitment [24–26], recruiting donors with rare HLA phenotypes [27], or alternatively using currently available HLA allele and haplotype frequencies [25, 28]. Although there is limited HLA diversity data for Southern Africans (reviewed in [29]), Africans are considered to be genetically diverse [19] as has been determined using multiple markers [30–32], including HLA [33]. Most HLA families that exist globally are found in African populations [34], further confirming genetic diversity in these populations.

In this study, we describe HLA allele and haplotype frequency data from 237 donors registered with the SABMR, which serves as the source of unrelated marrow donors in South Africa. Frequencies of HLA-A, HLA-B, HLA-C, HLA-DRB1, and HLA-DQB1 alleles and haplotypes were analyzed with the aim of developing a resource for disease association, anthropology, and evolutionary studies. Furthermore, these data will support models for population-specific vaccine development [35] and will improve donor recruitment strategies in South African populations.

## 2. Methods

### 2.1. Study Population, Data Access, and Ethics

Two hundred and thirty-seven (237) SABMR-registered-consenting volunteer bone marrow donors HLA typed at varying resolutions were included in this study. This subset was accessed following an extensive reconsenting procedure of donors in the SAMBR. The self-reported ethnic grouping of the study population was Asian, Black, Chinese, Coloured, White, and some unknown. High-resolution typing has recently been adopted by SABMR, with most donors having low-resolution typing (two digit) [20, 21] which did not meet the current study criteria. For ethical compliance, the current study had to reconsent donors to participate in the study. As a result, only 237 of the potential 400 participants provided consent. Ethical clearance for this study was granted by the University of Pretoria, Faculty of Health Sciences Research Ethics Committee (220/2015) and the SABMR Board. Participants' data accessed included HLA-A, HLA-B, HLA-C, HLA-DRB1, and HLA-DQB1 loci molecular typings and self-reported ethnicity. Some typings in this dataset were represented by multiple allele codes (MAC, formerly NMDP allele codes) as described in https://hml.nmdp.org/MacUI/.

### 2.2. HLA Allele and Haplotype Frequency Analysis

Allele and haplotype (two, three, four, and five loci) frequencies were estimated by resolving phase and allelic ambiguities using the expectation-maximization (EM) algorithm [36, 37] in Hapl-o-Mat open source software [38]. This software allows for allele verification using the IMTG/HLA database (http://www.ebi.ac.uk/ipd/imgt/hla/) [2, 3] and recognizes ambiguities including MACs. Deviations from Hardy-Weinberg equilibrium (HWE) were assessed at locus level using a chi-squared test [39]. Global linkage disequilibrium (LD) and HWE were implemented in Arlequin v3.5.2 [40]. MAC coded alleles were dropped to two-digit level resolution for HWE and LD analysis.

## 3. Results

### 3.1. Demographics and Allele Diversity

Self-reported ethnicity was not considered for analysis in this study owing to redundancy and simplicity of this classification as previously discussed [41, 42]. One hundred and thirty-one (131) Black, 69 Caucasian, 19 Mixed ancestry (Coloured), 15 Asian, 2 unknown, and 1 Chinese individuals were included in this study. Nine hundred and seventy-seven (977) different possible alleles are reported in this study (Table S1). There were 438 HLA-A, 235 HLA-B, 29 HLA-C, 234 HLA-DRB1, and 41 HLA-DQB1 alleles (Table S1), with the HLA-C locus having the lowest allelic diversity.

### 3.2. Hardy-Weinberg Equilibrium and Global LD Analysis

In this donor subset, HLA-A, HLA-B, and HLA-DRB1 genotypes deviated from the expected HWE proportions (*p* < 0.05), with HLA-C and HLA-DQB1 having insignificant (*p* > 0.05) differences between expected and observed heterozygosity (Table 1). No significant global LD was detected between A~B, A~C, B~C, A~DRB1, B~DRB1, C~DRB1, A~DQB1, B~DQB1, and DRB1~DQB1 locus pairs (Table 2). In addition, the C~DQB1 locus pair showed significant LD (*p* < 0.001), as summarized in Table 2.

### 3.3. HLA Allele Frequency

The full list of alleles including those derived from MACs and their frequencies are listed in Table S1. The top 20 most frequent alleles across the five loci are summarized in Table 3 with the top three alleles per locus being A^∗^02:01g (0.096), A^∗^03:01g (0.093), and A^∗^01:01g (0.057); B^∗^07:02g (0.082), B^∗^08:01g (0.049), and B^∗^58:02 (0.048); C^∗^07:02g (0.180), C^∗^07:01g (0.104), and C^∗^04:01g (0.091); DRB1^∗^15:01 (0.072), DRB1^∗^15:03 (0.065), and DRB1^∗^07:01 (0.057); and DQB1^∗^06:02 (0.157), DQB1^∗^03:01 (0.139), and DQB1^∗^05:01 (0.118).

### 3.4. HLA Haplotype Frequency

All two, three, four, and five (extended) haplotype frequencies are detailed in Supplementary Table 2 (Table S2), with the 20 most frequent haplotypes summarized in Tables 4 and 5 (extended haplotypes). The most common computed two, three, and four loci haplotypes were B∗07:02g~C∗07:02g (0.145), C∗07:02g~DRB1∗15:01~DQB1∗06:02 (0.107), and B∗07:02g~C∗07:02g~DRB1∗15:01~DQB1∗06:02 (0.108), respectively. We report a possible 7498 two locus, 6446 three locus, and 773 four locus haplotypes in the SABMR subset of the donors (Table s2). A^∗^33:95~B^∗^07:231N (1.08725E-06), A^∗^03:01g~C^∗^07:02g~DQB1^∗^03:02 (1.03519E-06), and A^∗^11:01g~C^∗^01:02g~DRB1^∗^01:01~DQB1^∗^05:01 (2.8507E-06) were less frequent two, three, and four locus haplotypes, respectively (Table S2). The twenty most frequent extended haplotypes (five loci) are summarized in Table 5, with A^∗^03:01g~B^∗^07:02g~C^∗^07:02g~DRB1^∗^15:01~DQB1^∗^06:02 being the most frequent (0.067).

## 4. Discussion

Although this study had a limited sample size of 237, we provide an in-depth analysis of HLA diversity in a subset of donors in the SABMR. Mixed resolution HLA typing data with multiple allele codes (https://hml.nmdp.org/MacUI) were analyzed using a robust Hapl-o-Mat [38] package to compute allele and haplotype frequencies through the EM algorithm. In addition, the package supports typing ambiguities in NMDP codes (MAC), G group and GL string formats. Since Hapl-o-Mat does not compute LD and HWE, we reduced all MAC-encoded typings in our data set to two digit resolution to estimate these parameters in Arlequin v3.5.2 [40]. Although there was the possibility of underestimation due to the loss of some allele information, global LD and HWE deviation is important in genetic studies.

Strong LD of C~DQB1 locus pairs (*p* < 0.001 in Table 2) in our study suggests limited chances of recombination between alleles from these loci in our population hence a greater chance of being inherited together. LD patterns of HLA or other genes may be used to infer evolutionary relatedness of populations [44]. Generally, individuals with haplotypes in LD are more likely to find haplomatches and strong LD is indicative of evolutionary relatedness of those alleles/loci. Carvalho and colleagues [45] report HLA-A, HLA-B, and HLA-DRB1 in HWE (*p* > 0.05), which contrasts to the significant deviation (*p* < 0.05) observed in the current study (Table 1). Sample size and mixed typing resolution in the current study may have affected HWE proportions. When there is no deviation from HWE, HLA data may be used to infer human peopling history in anthropological studies [46]. Furthermore, there is evidence of large HWE deviations influencing EM algorithm-based allele and haplotype frequency estimations [47]. It is thus important to note the sample size and mixed typing resolution limitations of the current study in interpreting HWE and LD analysis.

Taking into account the nature of the HLA data in the current study, we report 977 possible alleles (Table S1). HLA-C had the lowest number (29) of alleles compared to HLA-A (438 alleles) which had the highest. There are generally more reported HLA-B alleles in the HLA database [2, 3]. We note though that previously most registries routinely typed HLA-A, HLA-B, and HLA-DRB1 for new donors with few being typed for HLA-C and HLA-DQB1 [48]. This might explain the observed allele numbers in our study. There is an ever increasing number of alleles in the database (currently 17,874 in the IMTG/HLA database release 3.31) [2, 3], with South Africa contributing some unique alleles [49, 50].

HLA-A∗02:01g with a frequency of 9.6% in the current study has been reported in North West England Caucasians at a higher frequency of 28.9% [51]. This English study also reported B∗07:02g, C∗07:02g, and DRB1∗15:01 at frequencies of 15.3%, 15.6%, and 15.9%, respectively [51], compared to 8.2%, 18.0%, and 7.2% in the current study. It is important to note that the fifth most common allele in our study, namely, A∗30:02g (5% frequency in Table 3 and Table S1), is identical (exon 2 and 3 amino acid sequence) to a novel A∗30:02:01:03 allele previously reported in a SABMR donor [49]. HLA-DQB1∗06:02 (15.7%) has been observed at higher frequencies in previous studies in West Africans (30.8%) and Shona Zimbabweans (24.7%) and is lower in Kenyans (14.6%), Colombians (15.0%), and people from Papua New Guinea (15.0%) [26]. HLA-DRB1∗15:01 (7.2%) in the current study (Table 3) has been reported previously in South African populations at varying frequencies: 11.2% in Caucasians and 2.4% in Black Africans [26]. Additionally, DRB1∗15:01 had a 3.8% frequency in Inuit women [52], 11.65% in Chinese [53], and more than 50% in North Africans, Asians, people from Oceania, and Europeans [54].

The main thrust of our study has been the ability to estimate, with high confidence, haplotype frequencies from mixed resolution typings including MAC (https://hml.nmdp.org/MacUI) encoded alleles [38]. No record of the most frequent two, three, and four loci haplotypes reported in this study (Table 4 and Table S2) is found in the allele frequency database [2, 3, 55]. The most frequent (6.7%) extended haplotype A∗03:01g~B∗07:02g~C∗07:02g~DRB1∗15:01~DQB1∗06:02 has previously been reported amongst Chinese populations at varying frequencies (0.93–5.20%) [53] compared to our 6.7%. There is no record of this haplotype in African populations in the AFND allele frequency database [56]. A lower frequency (3.31%) of this haplotype has also been reported in a German registry as described by Sauter and colleagues [57].

Haplotype frequencies from a specific population may be useful for resolving typing ambiguities using statistical approaches in typing prospective individuals from the same population [58]. It is important though to note that sample size affects these computations, with a tendency towards haplotype overestimation in small sample-sized studies [35]. Other confounders include typing ambiguity as previously described [59]. Additionally, multilocus haplotype frequency estimation better informs disease association studies than allele frequency [47]. A complete list of donor registry HLA haplotype frequencies better informs donor-patient matching tools like EasyMatch® [60], NMDP HapLogic [61, 62], and OptiMatch [63] especially for patients of African origin who might benefit from donors in the SABMR. These tools use haplotype frequencies to compute the likelihood of a donor-patient match and also anticipate the most likely mismatches. Haplotype frequency may be used to estimate the probability of finding a recipient match or may give an indication of the likelihood of mismatches from initial registry searches [35]. Additionally, haplotypes are better indicators of HLA match estimation compared to allele frequency alone [35]. Variations in allele frequency distribution in populations in general provide insight into peopling history [64, 65]. HLA genetic makeup of populations provides insight into history including selective pressures by pathogens [33], migration, admixture, and changes in population size [54, 66–68].

Allele and haplotype frequencies from this study highlight the need for continued analysis by the SABMR for a better understanding of HLA diversity in the region. There is limited HLA diversity data for South African populations (reviewed in [29]), despite the evident value in transplantation, donor recruitment, disease association, and population studies. In addition, some registries specifically aim to improve recruitment from ethnic minorities [25] to increase the HLA diversity and hence the probability of finding an appropriate donor for a given patient. In this context, knowledge of the distribution of alleles and haplotypes in many different population groups, as determined by high-resolution typing, may allow for modification of recruitment strategies.

## 5. Conclusions

Although results reported here are from a small subset of SABMR registered donors, allele and haplotype frequencies generated by Hapl-o-Mat tool [38] could be a useful resource for future anthropological and population genetics studies in South Africans. Furthermore, these findings may better inform donor recruitment strategies for the SABMR. The small sample size limitation of this study also highlights the need for larger studies in order to better understand HLA diversity in South African populations. It would also be interesting to analyze the whole donor registry and compare its HLA diversity data to other registries globally.

## Figures and Tables

**Table 1:** Hardy-Weinberg equilibrium (HWE) parameters for the 237 donors studied.

Locus	Obs Het	Exp Het	SD	Steps done	*p* value
HLA-A	1.00000	0.96196	0.00000	1001000	<0.001^∗^
HLA-B	0.99554	0.97382	0.00001	1001000	0.00074^∗^
HLA-C	1.00000	0.93582	0.00020	1001000	0.07316
HLA-DRB1	0.98958	0.95618	0.00000	1001000	<0.001^∗^
HLA-DQB1	1.00000	0.91336	0.00027	1001000	0.15049

SD: standard deviation; ^∗^Statistically significant (*p* < 0.005).

**Table 2:** Pair-wise global LD estimates across the five loci.

Haplotype	Chi-square test value	Degrees of freedom	*p* value
A~B	1672.062	3696	1.000
A~C	845.290	1488	1.000
B~C	1220.641	2387	1.000
A~DRB1	1288.195	2256	1.000
B~DRB1	1713.476	3619	1.000
C~DRB1	847.773	1457	1.000
A~DQB1	596.485	816	1.000
B~DQB1	777.193	1309	1.000
C~DQB1	732.281	527	<0.001^∗^
DRB1~DQB1	802.780	799	0.456

^∗^Statistically significant (*p* < 0.005).

**Table 3:** The twenty most frequent HLA-A, HLA-B, HLA-C, HLA-DRB1, and HLA-DQB1 alleles from the 237 donor subset (full list in Table S1).

A	Frequency	B	Frequency	C	Frequency	DRB1	Frequency	DQB1	Frequency
A∗02:01g	0.096	B∗07:02g	0.082	C∗07:02g	0.180	DRB1∗15:01	0.072	DQB1∗06:02	0.157
A∗03:01g	0.093	B∗08:01g	0.049	C∗07:01g	0.104	DRB1∗15:03	0.065	DQB1∗03:01	0.139
A∗01:01g	0.057	B∗58:02	0.048	C∗04:01g	0.091	DRB1∗07:01	0.057	DQB1∗05:01	0.118
A∗24:02g	0.051	B∗42:01	0.039	C∗06:02g	0.074	DRB1∗13:01	0.053	DQB1∗02:01	0.090
A∗30:02g	0.050	B∗44:03	0.033	C∗08:02g	0.057	DRB1∗11:01	0.053	DQB1∗03:02	0.083
A∗68:02g	0.048	B∗15:10	0.032	C∗02:02g	0.051	DRB1∗03:01	0.046	DQB1∗06:03	0.068
A∗11:01g	0.044	B∗15:01g	0.031	C∗15:02g	0.045	DRB1∗04:01	0.038	DQB1∗04:02	0.066
A∗30:01g	0.043	B∗15:03g	0.031	C∗05:01g	0.045	DRB1∗03:02	0.034	DQB1∗02:02	0.063
A∗29:02g	0.035	B∗35:01g	0.031	C∗03:04g	0.045	DRB1∗13:02	0.033	DQB1∗05:03	0.049
A∗23:01g	0.034	B∗14:02	0.028	C∗12:03g	0.040	DRB1∗01:02	0.029	DQB1∗03:03	0.045
A∗68:01g	0.025	B∗58:01g	0.028	C∗03:03g	0.034	DRB1∗01:01	0.029	DQB1∗06:01	0.042
A∗43:01	0.024	B∗18:01g	0.026	C∗01:02g	0.034	DRB1∗15:02	0.026	DQB1∗03:19	0.021
A∗66:01g	0.023	B∗51:01g	0.025	C∗17:01g	0.028	DRB1∗11:02	0.021	DQB1∗06:04	0.021
A∗33:03g	0.023	B∗15:16	0.021	C∗12:02g	0.028	DRB1∗13:03	0.020	DQB1∗06:09	0.021
A∗34:02	0.022	B∗13:02g	0.021	C∗16:01g	0.023	DRB1∗11:04	0.018	DQB1∗04:04	0.003
A∗74:01g	0.020	B∗58:60	0.019	C∗14:02g	0.023	DRB1∗12:01	0.016	DQB1∗03:30	0.003
A∗31:01g	0.020	B∗53:01g	0.018	C∗18:01g	0.017	DRB1∗12:02	0.015	DQB1∗06:40	0.003
A∗24:07	0.017	B∗45:01g	0.018	C∗08:04	0.017	DRB1∗08:04	0.014	DQB1∗06:11	0.003
A∗02:05g	0.016	B∗81:01g	0.018	C∗07:04g	0.017	DRB1∗14:04	0.013	DQB1∗06:218	0.000
A∗33:01g	0.015	B∗27:05g	0.017	C∗03:02g	0.017	DRB1∗03:102	0.013	DQB1∗06:185	0.000

g: groups are expressed and null alleles with identical amino acid sequences across class I exons 2 and 3 and class II exon 2 [43].

**Table 4:** The twenty most frequent two, three, and four locus haplotype frequencies in the 237 donor subset (Full list in Table S2).

Two loci	Freq	Three loci	Freq	Four loci	Freq
B∗07:02g~C∗07:02g	0.145	C∗07:02g~DRB1∗15:01~DQB1∗06:02	0.107	B∗07:02g~C∗07:02~DRB1∗15:01g~DQB1∗06:02	0.108
DRB1∗15:01~DQB1∗06:02	0.125	B∗07:02g~DRB1∗15:01~DQB1∗06:02	0.106	B∗08:01g~C∗07:01g~DRB1∗03:01~DQB1∗02:01	0.067
C∗07:02g~DQB1∗06:02	0.105	B∗07:02g~C∗07:02g~DQB1∗06:02	0.101	A∗03:01g~B∗07:02g~C∗07:02g~DQB1∗06:02	0.063
B∗07:02g~DQB1∗06:02	0.099	B∗07:02g~C∗07:02g~DRB1∗15:01	0.084	A∗03:01g~B∗07:02g~DRB1∗15:01~DQB1∗06:02	0.061
DRB1∗03:01~DQB1∗02:01	0.096	A∗03:01g~B∗07:02g~C∗07:02g	0.081	A∗03:01g~C∗07:02g~DRB1∗15:01~DQB1∗06:02	0.057
C∗07:02g~DRB1∗15:01	0.091	B∗08:01g~DRB1∗03:01~DQB1∗02:01	0.076	A∗03:01g~B∗07:02g~C∗07:02g~DRB1∗15:01	0.051
A∗03:01g~C∗07:02g	0.079	C∗07:01g~DRB1∗03:01~DQB1∗02:01	0.066	A∗01:01g~C∗07:01g~DRB1∗03:01~DQB1∗02:01	0.049
B∗08:01g~DQB1∗02:01	0.071	B∗08:01g~C∗07:01g~DQB1∗02:01	0.063	A∗01:01g~B∗08:01g~C∗07:01g~DQB1∗02:01	0.047
DRB1∗13:01~DQB1∗06:03	0.071	A∗03:01g~C∗07:02g~DQB1∗06:02	0.062	A∗01:01g~B∗08:01g~DRB1∗03:01~DQB1∗02:01	0.045
A∗03:01g~DQB1∗06:02	0.061	A∗03:01g~B∗07:02g~DQB1∗06:02	0.057	A∗01:01g~B∗08:01g~C∗07:01g~DRB1∗03:01	0.037
B∗08:01g~C∗07:01g	0.058	B∗08:01g~C∗07:01g~DRB1∗03:01	0.052	B∗15:01g~C∗03:03g~DRB1∗13:01~DQB1∗06:03	0.029
C∗07:01g~DQB1∗02:01	0.053	A∗03:01g~DRB1∗15:01~DQB1∗06:02	0.051	B∗44:02g~C∗05:01g~DRB1∗01:01~DQB1∗05:01	0.025
DRB1∗01:01~DQB1∗05:01	0.051	A∗01:01g~B∗08:01g~C∗07:01g	0.047	A∗11:01g~C∗01:02g~DRB1∗15:01~DQB1∗06:02	0.025
DRB1∗11:01~DQB1∗03:01	0.051	A∗01:01g~C∗07:01g~DQB1∗02:01	0.046	A∗03:01g~C∗07:02g~DRB1∗01:01~DQB1∗05:01	0.025
C∗07:01g~DRB1∗03:01	0.049	A∗03:01g~C∗07:02g~DRB1∗15:01	0.044	A∗03:01g~B∗07:02g~C∗07:02g~DQB1∗03:01	0.023
C∗04:01g~DQB1∗05:01	0.045	A∗01:01g~B∗08:01g~DQB1∗02:01	0.043	A∗11:01g~B∗51:01g~DRB1∗15:01~DQB1∗06:02	0.023
DRB1∗07:01~DQB1∗02:02	0.044	A∗01:01g~DRB1∗03:01~DQB1∗02:01	0.042	A∗02:01g~B∗07:02g~DRB1∗15:01~DQB1∗06:02	0.023
B∗07:02g~DRB1∗15:01	0.043	A∗01:01g~C∗07:01g~DRB1∗03:01	0.037	B∗42:01~C∗17:01g~DRB1∗03:02~DQB1∗04:02	0.021
A∗01:01g~DQB1∗02:01	0.042	A∗11:01g~DRB1∗13:01~DQB1∗06:03	0.037	B∗57:01g~C∗06:02g~DRB1∗07:01~DQB1∗03:03	0.021
B∗14:02~C∗08:02g	0.041	A∗24:02g~B∗07:02g~C∗07:02g	0.035	A∗01:01g~C∗06:02g~DRB1∗07:01~DQB1∗03:03	0.020

Freq: frequency; g: groups are expressed and null alleles with identical amino acid sequences across class I exons 2 and 3 and class II exon 2 [43].

**Table 5:** The twenty most frequent extended (five loci) haplotype frequencies from the 237 donor subset in the SABMR (full list in Table S2).

A~B~C~DQB1~DRB1 haplotype	Frequency
A∗03:01g~B∗07:02g~C∗07:02g~DRB1∗15:01~DQB1∗06:02	0.067
A∗01:01g~B∗08:01g~C∗07:01g~DRB1∗03:01~DQB1∗02:01	0.050
A∗01:01g~B∗57:01g~C∗06:02g~DRB1∗07:01~DQB1∗03:03	0.021
A∗03:01g~B∗07:02g~C∗07:02g~DRB1∗01:01~DQB1∗05:01	0.017
A∗11:01g~B∗15:01g~C∗03:03g~DRB1∗13:01~DQB1∗06:03	0.017
A∗24:02g~B∗07:02g~C∗07:02g~DRB1∗15:01~DQB1∗06:02	0.017
A∗02:11g~B∗40:06~C∗15:02g~DRB1∗15:01~DQB1∗06:01	0.017
A∗33:01g~B∗14:02~C∗08:02g~DRB1∗13:01~DQB1∗06:03	0.017
A∗68:02g~B∗14:01~C∗08:02g~DRB1∗07:01~DQB1∗02:02	0.017
A∗11:01g~B∗51:01g~C∗01:02g~DRB1∗04:01~DQB1∗03:02	0.017
A∗31:01g~B∗27:05g~C∗02:02g~DRB1∗15:01~DQB1∗06:02	0.017
A∗03:01g~B∗07:02g~C∗07:02g~DRB1∗11:01~DQB1∗03:01	0.017
A∗68:02g~B∗14:02~C∗08:02g~DRB1∗13:03~DQB1∗03:01	0.017
A∗69:01~B∗15:17~C∗07:01g~DRB1∗11:01~DQB1∗03:01	0.017
A∗02:01g~B∗07:02g~C∗07:02g~DRB1∗15:01~DQB1∗06:02	0.017
A∗30:01g~B∗42:01~C∗17:01g~DRB1∗03:02~DQB1∗04:02	0.013
A∗24:02g~B∗15:32~C∗12:03g~DRB1∗12:02~DQB1∗03:01	0.008
A∗23:01g~B∗49:01g~C∗07:01g~DRB1∗15:02~DQB1∗05:03	0.008
A∗25:01g~B∗08:01g~C∗07:01g~DRB1∗03:01~DQB1∗02:01	0.008
A∗26:01g~B∗58:01g~C∗05:01g~DRB1∗15:03~DQB1∗06:02	0.008

g: groups are expressed and null alleles with identical amino acid sequences across class I exons 2 and 3 and class II exon 2 [43].

## Supplementary Materials

Supplementary Materials Supplementary Table 1 (Table S1): HLA-A, HLA-B, HLA-C, HLA-DRB1, and HLA-DQB1 allele frequencies in 237 volunteer bone marrow donors registered in the South African Bone Marrow Registry. The 237 individuals described herein are a subset of all SABMR registered donors. Supplementary Table 2 (Table S2): Two, three, four, and five loci Haplo-o-Mat [38] estimated haplotype frequencies in 237 volunteer bone marrow donors registered in the South African Bone Marrow Registry. The 237 individuals described herein are a subset of all SABMR registered donors.
